# A Low-Cost Cocoa-Opacified Hematemesis Simulant for Manikin-Based Airway and Endoscopic Visualization Training: Formulation Rationale, Targeted Review, and Cost Analysis

**DOI:** 10.7759/cureus.107966

**Published:** 2026-04-29

**Authors:** Bailey L Jarrett, Kevin T Malone, Lois Rotuno, Dorian F Drigalla, Andrew L Juergens II

**Affiliations:** 1 Emergency Medicine, Baylor College of Medicine, Temple, USA; 2 Emergency Medicine, Baylor Scott & White Medical Center, Temple, USA

**Keywords:** blood, hematemesis, opacity, simulation, video

## Abstract

High-fidelity manikin scenarios for contaminated airway management and upper gastrointestinal bleeding (UGIB) depend on blood or hematemesis simulants that reliably occlude optics and reproduce the visual gestalt of real blood under high-intensity video laryngoscopy and flexible endoscopy lighting. Commercially available simulated blood products are frequently optimized for reusability, stain resistance, and compatibility with procedural trainers, and can be cost-prohibitive for repeated high-volume “soiling” curricula. However, many simulated blood products do not report optical opacity or camera-obscuration performance for this specific use case.

We performed a targeted technical review of blood and bleeding simulant strategies used in manikin-based training and adjacent materials science literature, and we describe a low-cost, reproducible hematemesis analog composed of three packets of a raspberry-flavored powdered beverage mix - Crystal Light Sugar-Free Raspberry Ice Drink Mix (made from maltodextrin, citrate salts, calcium phosphate, organic acids, and FD&C dyes) - dispersed with 20 g of unsweetened cocoa powder - Hershey's Cocoa - in 1 L of water using an immersion blender. In preliminary qualitative bench use, this particulate suspension formulation produced a dark red-brown appearance and visually reduced light transmission compared with the dye-only beverage mixture. Objective optical, rheologic, and comparative performance testing was not performed in this introductory report. Direct material cost was approximately $1.66/L using representative U.S. retail pricing, compared with $7-$29/L for selected commercial powders and premixes, although this comparison reflects direct consumable cost only and does not establish performance equivalence. Limitations include sedimentation and phase separation over time (necessitating re-homogenization) and potential staining of the manikin; a spot test on non-critical surfaces is recommended prior to implementation. Because the formulation contains organic particulates, it should not be introduced into patient-care endoscope working channels or equipment intended for later clinical use unless local reprocessing staff verify complete clearance for the specific device. This report is intended as a preliminary formulation and microcosting analysis; future work should quantify viscosity (including shear-thinning behavior), density, spectral absorbance/scattering, and material compatibility to support standardization across simulation programs.

## Introduction

Training for contaminated airway management and upper gastrointestinal bleeding (UGIB) is fundamentally a visualization problem: the operator must establish and maintain a view through a narrow optical channel while suctioning, manipulating tissues, and coordinating team actions under time pressure. The SALAD (Suction-Assisted Laryngoscopy Assisted Decontamination) paradigm and related curricula have demonstrated that deliberate practice with active regurgitation/bleeding simulators can be implemented using standard manikins and inexpensive adjuncts [1,2]. Prospective and observational studies have used contaminated airway simulation to teach suction technique, optimize approach selection, and improve performance metrics in scenarios involving massive emesis or blood [3,4]. In actual clinical UGIB, the presence of blood and particulate gastric contents can reduce first-pass success and increase the technical difficulty of laryngoscopy and endoscopy, underscoring the need for realistic obscuration in training environments [5]. Structured simulation cases for acute UGIB and hematemesis have been described for learners across disciplines; however, the choice of “blood” is often treated as a practical afterthought rather than a controlled variable with measurable optical and rheologic properties [6].

Commercial simulated blood products are widely used for venipuncture trainers, wound moulage, and casualty simulation, but they are not uniformly designed for repeated high-volume “soiling” applications in which a realistic fluid must rapidly obscure a camera-based laryngoscope or a flexible endoscope lens. In these use cases, insufficient opacity and/or insufficient particulate load may yield unrealistically translucent images under light-emitting diode (LED) illumination. The economic barrier is non-trivial: high-throughput simulation curricula (e.g., repeated SALAD drills across multiple learner cohorts) can require tens to hundreds of liters per year, making per-liter costs a first-order constraint on curriculum design and equity of access [7]. Accordingly, there is a practical need for a low-cost, reproducible hematemesis simulant made from easily obtainable materials, with optical opacity that better approximates real blood and blood-gastric content mixtures during endoscopic and airway procedures. This would not only help with medical training but also allow for the replicability of studies. Opacity is emphasized because camera-based laryngoscopes and endoscopes use intense local illumination; a red dye solution may reproduce color while still permitting unrealistic transillumination and residual visualization. Blood and blood-gastric mixtures also scatter light, foul lenses, and can abruptly eliminate the optical field. Published simulation reports rarely characterize these optical endpoints quantitatively, which is why the present article is framed as an introductory formulation and cost analysis rather than a validation study.

The remaining formulation problem is therefore not whether a simulant appears red in a static container, but whether it predictably degrades the optical field during camera-based airway and endoscopic tasks. Prior contaminated airway, SALAD, UGIB, and hematemesis simulation literature supports the educational need for active soiling models, but these reports generally emphasize technique, learner performance, or case design rather than controlled measurement of the simulant’s optical behavior [1-6]. This distinction matters because videolaryngoscopes and flexible endoscopes use high-intensity local illumination; dye-only fluids may reproduce hue while remaining unrealistically translucent, whereas real blood functions as a dense cellular suspension with wavelength-dependent absorption and strong light scattering [8]. The problem also has a practical scale component, because repeated soiling curricula may require large fluid volumes, making per-liter cost a meaningful constraint on implementation and equitable access [7]. For this reason, adjacent materials science, forensic, and low-cost simulator literature is useful: these fields treat blood analogs as engineered materials with tunable optical, rheologic, and stability properties rather than as cosmetic red fluids [9,10]. A practical hematemesis simulant must therefore balance chromophore-based color, particulate scattering, cost, reproducibility, and the predictable tradeoff of sedimentation requiring re-homogenization.

This article used a targeted technical review rather than a systematic review. We searched PubMed, MedEdPORTAL, Google Scholar, and publicly available manufacturer/distributor materials for sources addressing contaminated airway simulation, SALAD training, UGIB simulation, optical properties of blood, engineered or forensic blood substitutes, and commercially available simulated blood products. Search terms included “simulated blood,” “hematemesis simulation,” “contaminated airway simulation,” “SALAD airway,” “blood optical properties,” “blood substitute,” “turbidity,” and “endoscopic simulation.” We prioritized sources that described training use of blood/emesis simulants, optical or rheologic properties relevant to visualization, or cost/product specifications relevant to high-volume simulation. Because objective optical performance data for manikin blood simulants under videolaryngoscope or endoscope lighting are sparse, this review was used to define design requirements rather than to perform a pooled performance comparison.

## Technical report

Design requirements for visualization-focused blood and hematemesis simulants

For training tasks where the principal outcome is visualization (rather than ultrasound acoustic fidelity), the most important performance parameters are optical absorption and scattering, lens fouling behavior, and perceptual realism under the relevant lighting geometry (videolaryngoscope LEDs, endoscopic light sources, and headlamps). Whole blood is a concentrated suspension of cells and proteins with strong wavelength-dependent absorption and substantial scattering driven primarily by erythrocyte morphology and concentration [8]. Realistic simulants therefore require more than “red dye in water”: in addition to chromophore selection, a formulation must generate sufficient turbidity (optical density) to prevent unrealistic transillumination and to reproduce the abrupt loss of a view that occurs when blood floods an optical field.

Hematemesis adds additional constraints. Clinically, UGIB fluid is often heterogeneous, containing blood, gastric secretions, and particulate matter; visually, it may range from bright red to dark maroon or coffee-ground material depending on digestion, dilution, and clot burden. For manikin-based UGIB and airway contamination simulation, a useful hematemesis analog should therefore achieve a dark red-brown tone, include a stable or re-suspendable particulate fraction that creates light scattering, and maintain handling properties compatible with suction devices and manikin plumbing. At the same time, a simulant must be safe, inexpensive, easily cleaned, and sufficiently reproducible to be shared across institutions and study sites.

Formulation strategies for blood and bleeding simulants

Across the simulation literature and adjacent materials science domains, blood simulants can be grouped into four broad formulation strategies: dye-based aqueous solutions (chromophore-dominant), thickened solutions (rheology-dominant), particulate suspensions (scattering-dominant), and reactive systems that form clots when exposed to specific training dressings or triggers (coagulation-analog systems). In practice, many commercial and home-built recipes use hybrid approaches to balance color, opacity, viscosity, stability, and cleanup. The forensic pattern-analysis literature is instructive because it explicitly treats “blood” as a material with tunable properties (surface tension, viscosity, density, and optical appearance) rather than as a cosmetic effect [9].

Dye-based aqueous solutions are inexpensive and easy to clean, but they are frequently too transparent under endoscopic or video laryngoscopic illumination and may fail to reproduce the visual extinction caused by whole blood. Thickened solutions, often achieved using hydrocolloids or cellulose derivatives, improve “pour” and can introduce non-Newtonian behavior; they also slow particulate settling and can increase lens fouling by increasing residence time on the optic. In contaminated airway simulation specifically, published SALAD implementations have used thickened “vomitus” formulations (e.g., vinegar combined with xanthan gum) to create continuous soiling during laryngoscopy [1]. Related task-trainer modifications have emphasized low-cost pumps and plumbing to deliver contaminants, but recipes and quantitative material specifications remain heterogeneous [4].

Particulate suspensions are the most direct route to high optical opacity. By dispersing fine particles that induce Mie scattering, a suspension can rapidly obscure a camera lens and eliminate unrealistic transillumination/visualization, even when the total dissolved dye concentration is modest. Cocoa, starches, and other food-grade powders are readily available opacifiers; engineering and forensic approaches have used controlled particle systems and polymer networks to tune stability and flow [9]. Low-cost bleeding simulators in procedural training frequently rely on the same principle - using inexpensive pumps and reservoir systems with surrogate blood - though detailed reporting of the surrogate fluid’s optical and rheologic targets is uncommon [10].

Coagulation-analog systems represent a different design goal: rather than purely obscuring optics, they aim to replicate clot formation and hemostatic device interactions, which are relevant for hemorrhage control and endoscopic hemostasis simulation. A practical example in endoscopy is the development of bleeding simulators that permit the use of actual endoscopes and hemostatic devices for procedural rehearsal [11]. These systems can be valuable but are typically costlier and may not be necessary when the training endpoint is primarily visual obstruction and suction mechanics rather than device-mediated clot behavior. The major simulation domains, the aspects of realism that matter most in each, and the corresponding implications for simulant design are summarized in Table 1.

**Table 1:** Blood and hematemesis simulation in manikins. Table credits: Kevin T. Malone. Sources [1-10]. SALAD: Suction-Assisted Laryngoscopy Assisted Decontamination; UGIB: upper gastrointestinal bleeding

Domain/scenario	What “realism” hinges on	Simulant implication
Soiled airway/SALAD-style laryngoscopy	Rapid loss of view; lens fouling; suction choreography under continuous contamination	Requires high scattering/opacity + film persistence; dye-only is too translucent [1-4,8].
UGIB/hematemesis airway management	Contaminated airway; impaired visualization as a pathway to difficulty/complications	Simulant should reproduce dark red-brown appearance and particulate heterogeneity consistent with hematemesis [5,6].
High-throughput curricula and multi-site protocols	Consumable cost and supply chain determine repetitions and feasibility	Recipe should be low-cost, reproducible, and made from readily obtainable materials with minimal vendor lock-in [7,10].
Engineered “blood substitutes” (materials/forensic adjacent)	Tunable optical + flow properties, not just color	Report optical + rheologic targets and accept stability tradeoffs (sedimentation) as a design variable [8,9].

Commercial blood simulants: typical design priorities and gaps

Commercial products span premixed fluids, concentrates, and powders. Premixed simulated blood is convenient but commonly priced for low-volume moulage or skills-trainer use rather than for continuous soiling drills. For example, simulated blood is marketed in quart-scale units with claims of stain resistance and viscosity similar to real blood [12]. Other premixed products are sold in gallon-scale containers for skills training, but product descriptions rarely specify optical density under high-intensity LED illumination or performance as an obscurant for camera-based laryngoscopy and endoscopy [13].

Powdered simulated blood products reduce shipping weight and can be reconstituted in larger volumes, but their formulations are typically optimized for general classroom use and may prioritize ease of dissolution and washability over maximal opacity. Manufacturer guidance for some powders explicitly acknowledges the risk of permanent staining on selected materials, reinforcing the need for compatibility testing with specific manikin plastics, silicone components, and scope housings [14]. Comparable powders are available across vendors, including products marketed for casualty simulation manikins that yield approximately one gallon per packet [15].

At the high-fidelity end, specialized blood simulants are designed to interact with training hemostatic dressings and “clot” under specific conditions, supporting hemorrhage control simulation [16]. While valuable for hemostasis curricula, these products are priced substantially higher per liter than general simulated blood and may not address the specific training problem of optical obscuration during hematemesis or airway contamination.

Concentrated blood products can, in principle, reduce storage volume and allow dilution to the desired color intensity; however, the effective cost per liter varies markedly with dilution ratio and still remains high relative to grocery-grade materials [17]. Taken together, the market reflects a broad need for either purpose-built optical obscurants or openly reproducible recipes that can be prepared at scale, with clear reporting of optical realism, handling, and compatibility.

Proposed formulation: cocoa-opacified beverage mix hematemesis analog

We developed a low-cost hematemesis analog using widely available, shelf-stable grocery components. The formulation is prepared by combining three packets of a raspberry-flavored powdered beverage mix - Crystal Light Sugar-Free Raspberry Ice Drink Mix (ingredients: maltodextrin, citric acid, malic acid, aspartame, FD&C Red 40, sodium citrate, calcium phosphate, acesulfame potassium, FD&C Blue 1, and flavoring agents) - dispersed with 20 g of unsweetened cocoa powder - Hershey's Cocoa - in 1 L of water. An immersion blender is used to disperse cocoa particles and break up agglomerates, producing a uniform dark red-brown suspension.

From a formulation standpoint, the beverage mix supplies chromophores (Red 40 and Blue 1) to approximate the spectral “redness” of blood while maltodextrin increases dissolved solids and slightly increases viscosity, which can improve the perception of thickness and reduce rapid drainage from surfaces. Citrate salts and organic acids buffer the solution and contribute to ionic strength; calcium phosphate may add a fine particulate component that can further increase light scattering. The cocoa powder provides the dominant optical effect by introducing a high number density of particles that generate turbidity and mimic the heterogeneous appearance of blood mixed with gastric contents. On gross visual inspection, the final suspension produced a dark red-brown appearance compatible with blood simulation; no claim of equivalence to human blood is made.

During preliminary qualitative bench handling with videolaryngoscope and flexible fiberoptic lighting, the cocoa-containing suspension produced visible lens obscuration and reduced transillumination compared with the dye-only beverage mixture. These observations are intended as face-validity observations only; turbidity, spectral transmittance, optical density, viscosity, and formal camera-occlusion outcomes were not measured in this introductory report. The same particulate mechanism that improves realism also introduces a predictable limitation: the mixture separates over time. Cocoa particles sediment under gravity, leading to a clearer supernatant and a denser lower layer if the fluid is left standing. Re-homogenization by shaking or brief re-blending restores opacity, but workflows should anticipate this behavior, particularly for multi-station simulation days.

Material compatibility should be treated as a testable parameter rather than an assumption. FD&C dyes and fine particulates can stain certain polymers, porous plastics, and textiles. Although informal testing suggested limited staining on silicone components, staining risk is surface- and material-dependent; programs should perform a small-area spot test on a non-critical region of each manikin or accessory prior to first use and should validate that cleanup agents do not degrade the simulator. Because cocoa is an organic material, storage for extended periods can also increase odor and microbial growth; this formulation is best prepared fresh or stored briefly under refrigeration with appropriate handling precautions.

Simulant composition, preparation, and handling

Each 1 L batch of hematemesis simulant was prepared using 1 L of water, three single-serve packets of Crystal Light Sugar-Free Raspberry Ice Drink Mix, and 20 g of unsweetened cocoa powder (Hershey’s Cocoa), which served as the primary particulate opacifier. Representative label ingredients of the beverage mix included maltodextrin, citric acid, malic acid, aspartame (phenylalanine source), FD&C Red 40, natural and artificial flavoring agents, sodium citrate, calcium phosphate, acesulfame potassium, and FD&C Blue 1.

**Figure 1:**
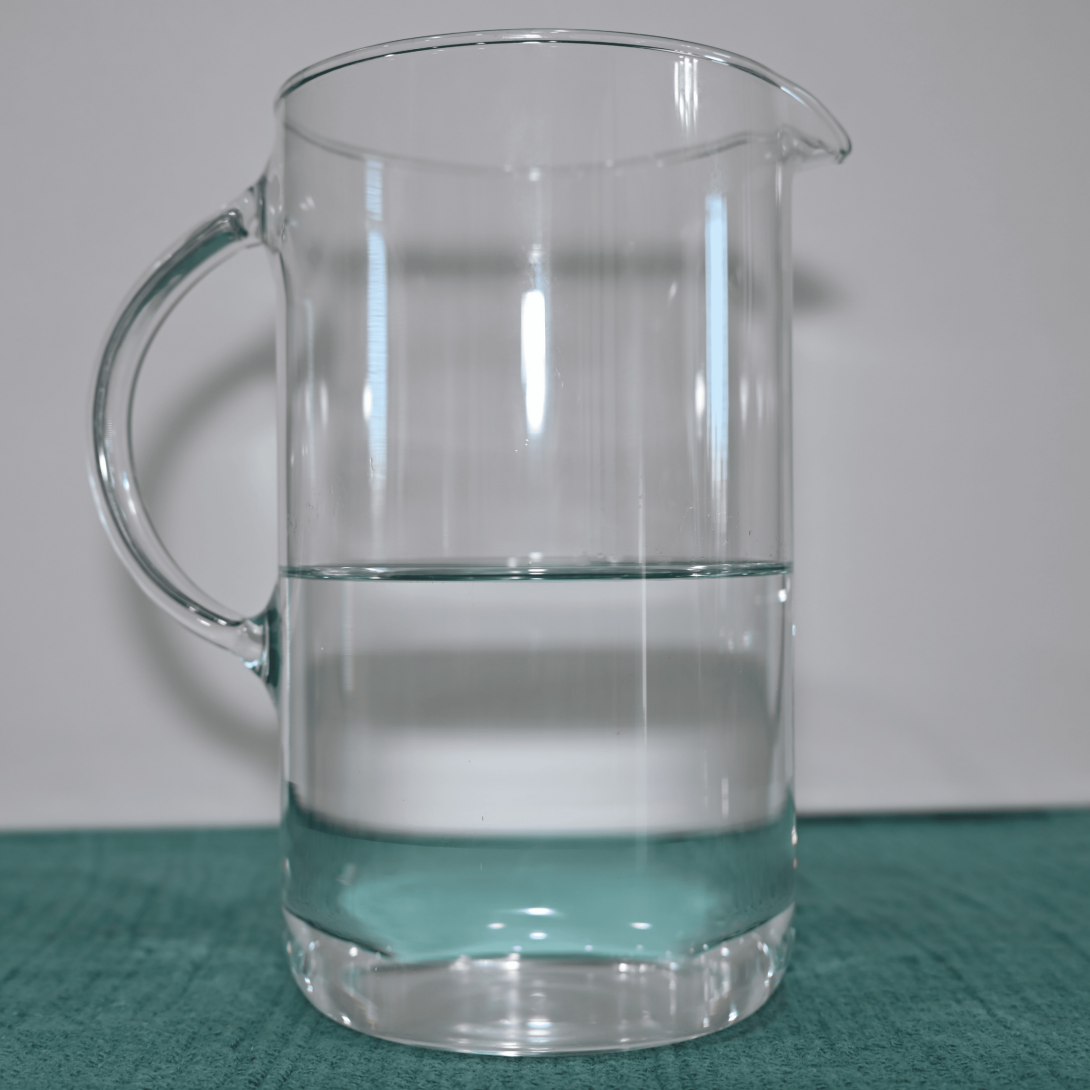
1 L of water, first step of the sequence.

**Figure 2:**
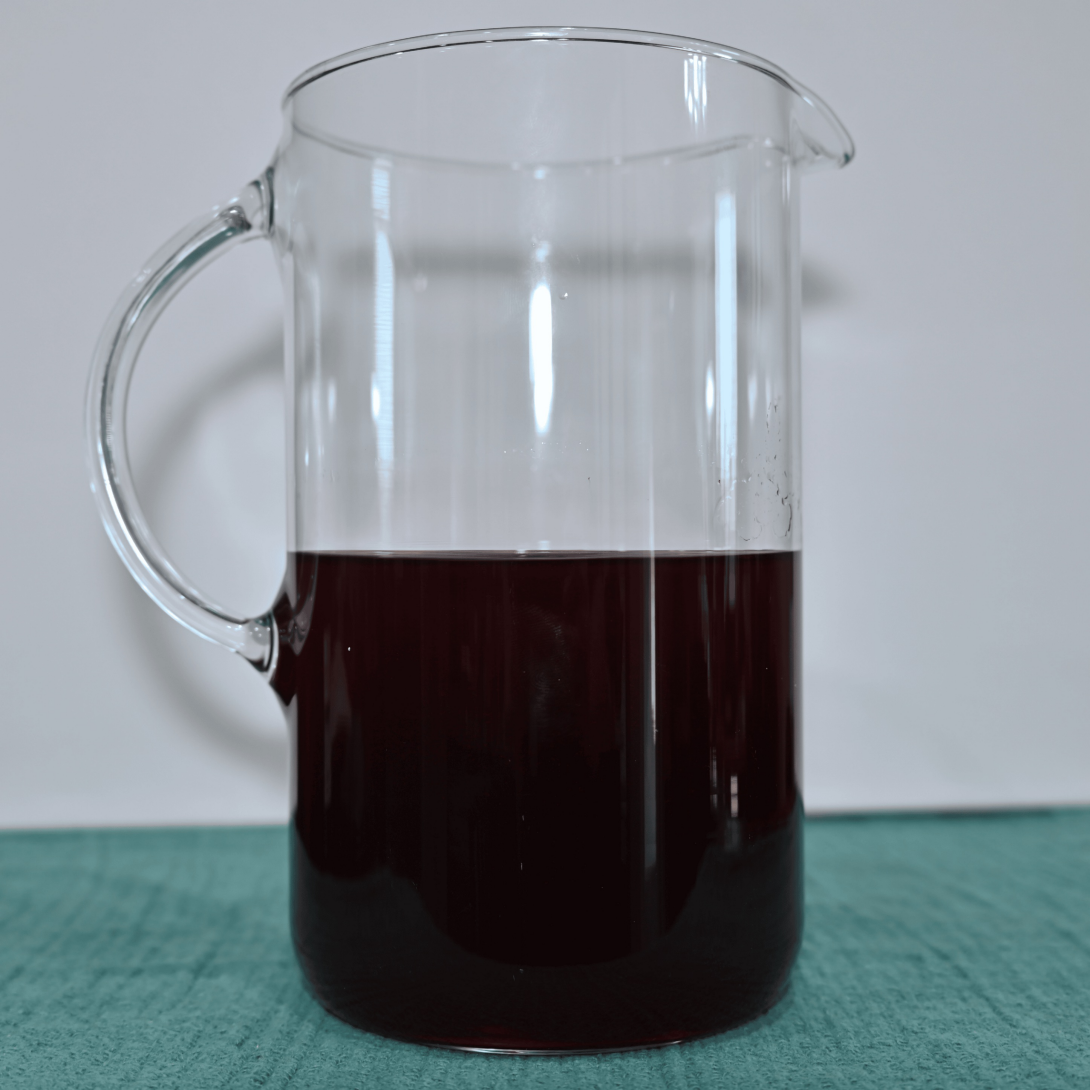
Mixture minus cocoa powder. Second step of the sequence.

**Figure 3:**
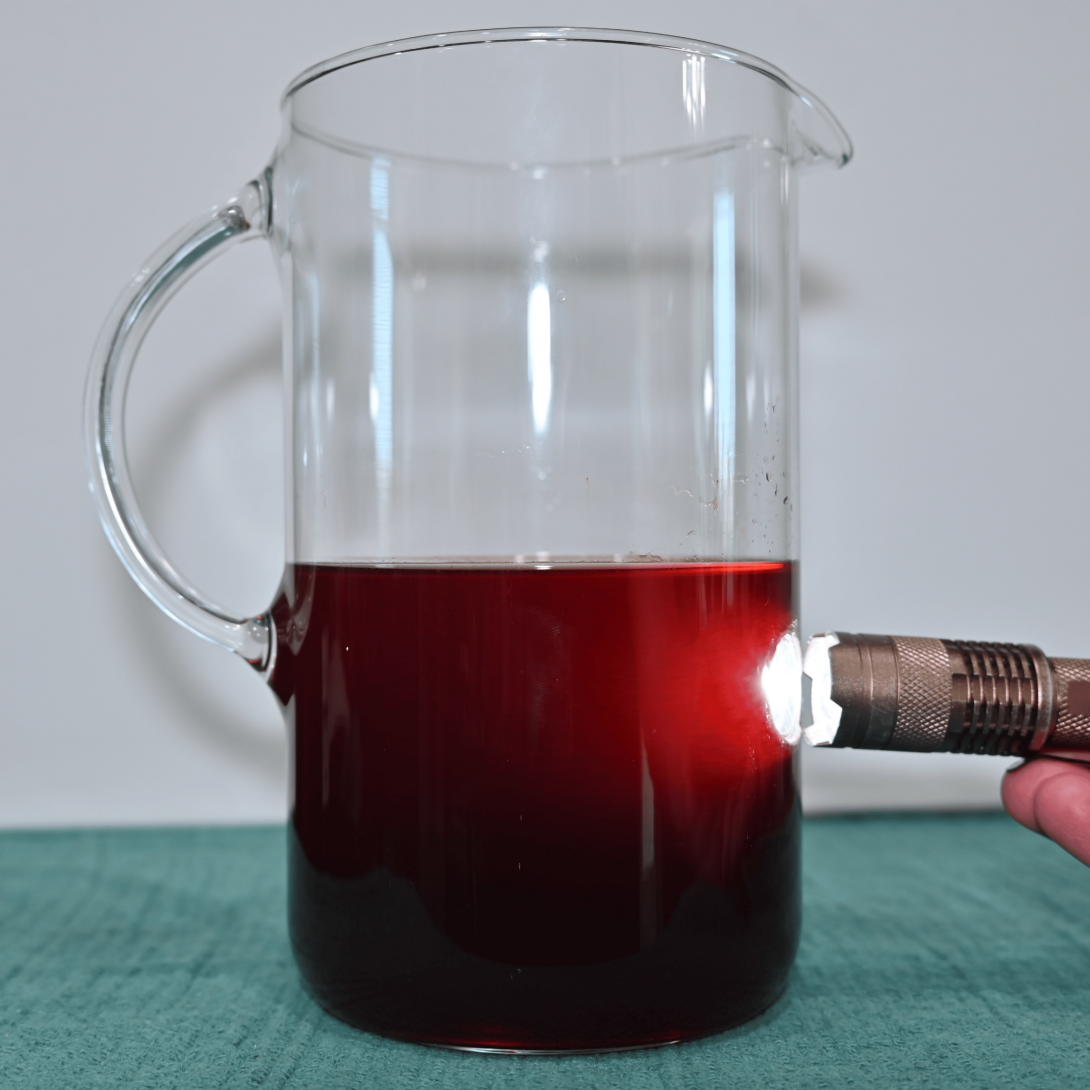
Mixture minus cocoa powder showing light penetration.

**Figure 4:**
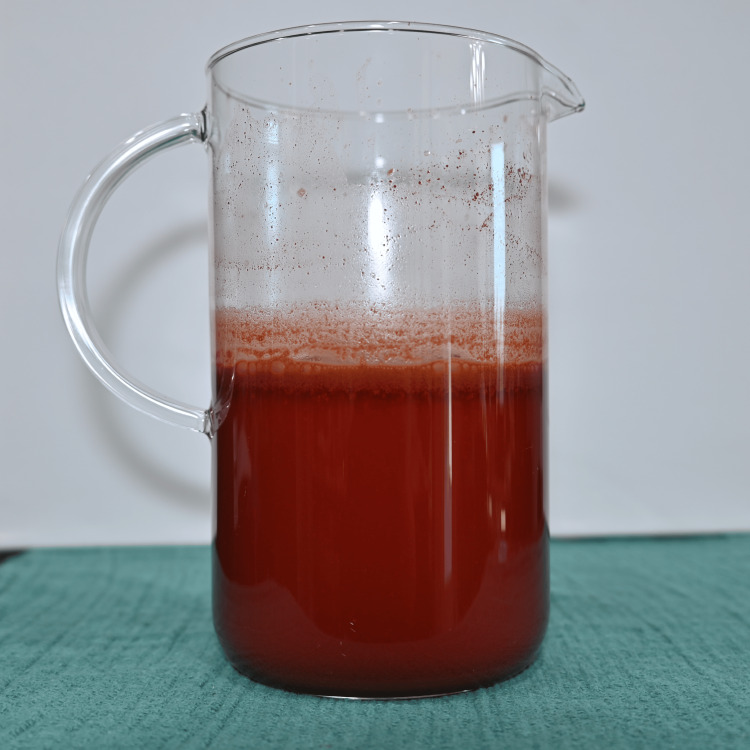
Mixture as described.

**Figure 5:**
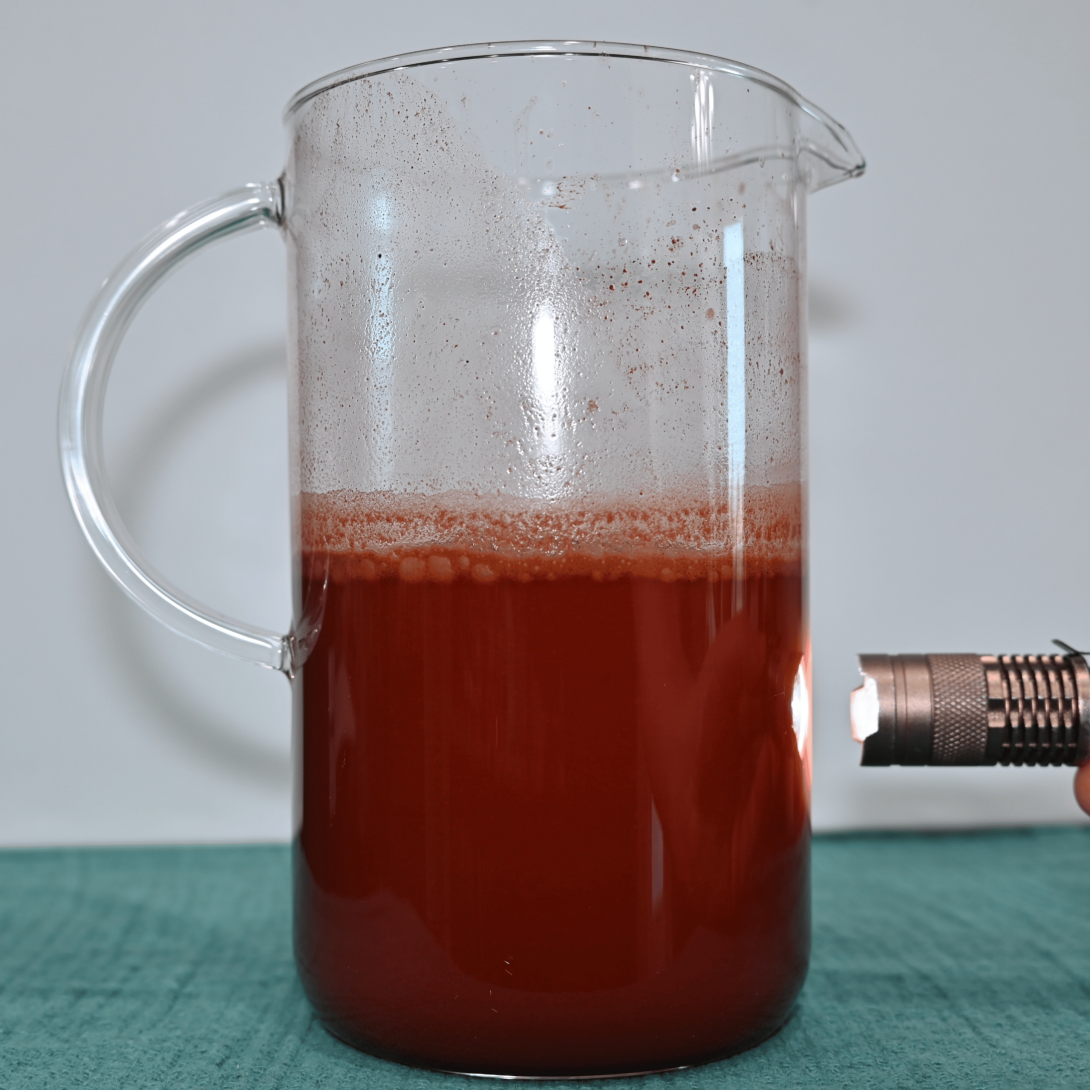
Mixture showing minimal light penetration.

Preparation was standardized to improve reproducibility. Water was first added to a mixing vessel as shown in Figure 1, followed by the beverage mix powder as seen in Figure 2, which was briefly blended to dissolve. As seen in Figure 3, light is able to penetrate this solution. Cocoa powder was then added gradually during low-speed blending to minimize agglomeration. Mixing was continued until the suspension appeared visually homogeneous, as seen in Figure 4. Note that in Figure 5, there is much less light able to penetrate the solution. An immersion blender was preferred because it improved the dispersion of cocoa particles and reduced clumping relative to hand mixing. Because cocoa sedimentation was expected over time, the mixture was either used immediately or kept covered and re-mixed before use. For prolonged use in reservoir-based or pump-driven simulation setups, periodic agitation was helpful to maintain uniform opacity.

**Figure 6:**
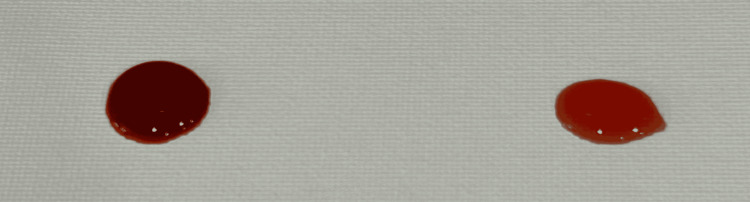
Comparison of full mixture (left) versus mixture minus cocoa powder (right).

**Figure 7:**
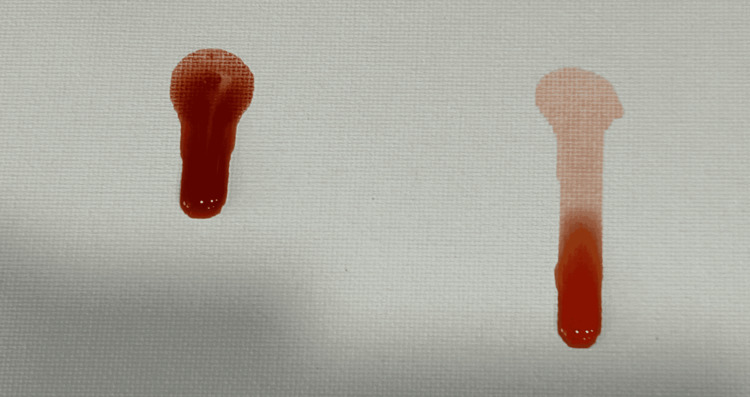
Demonstration of gross droplet appearance and spread of full mixture (left) versus mixture minus cocoa powder (right).

**Figure 8:**
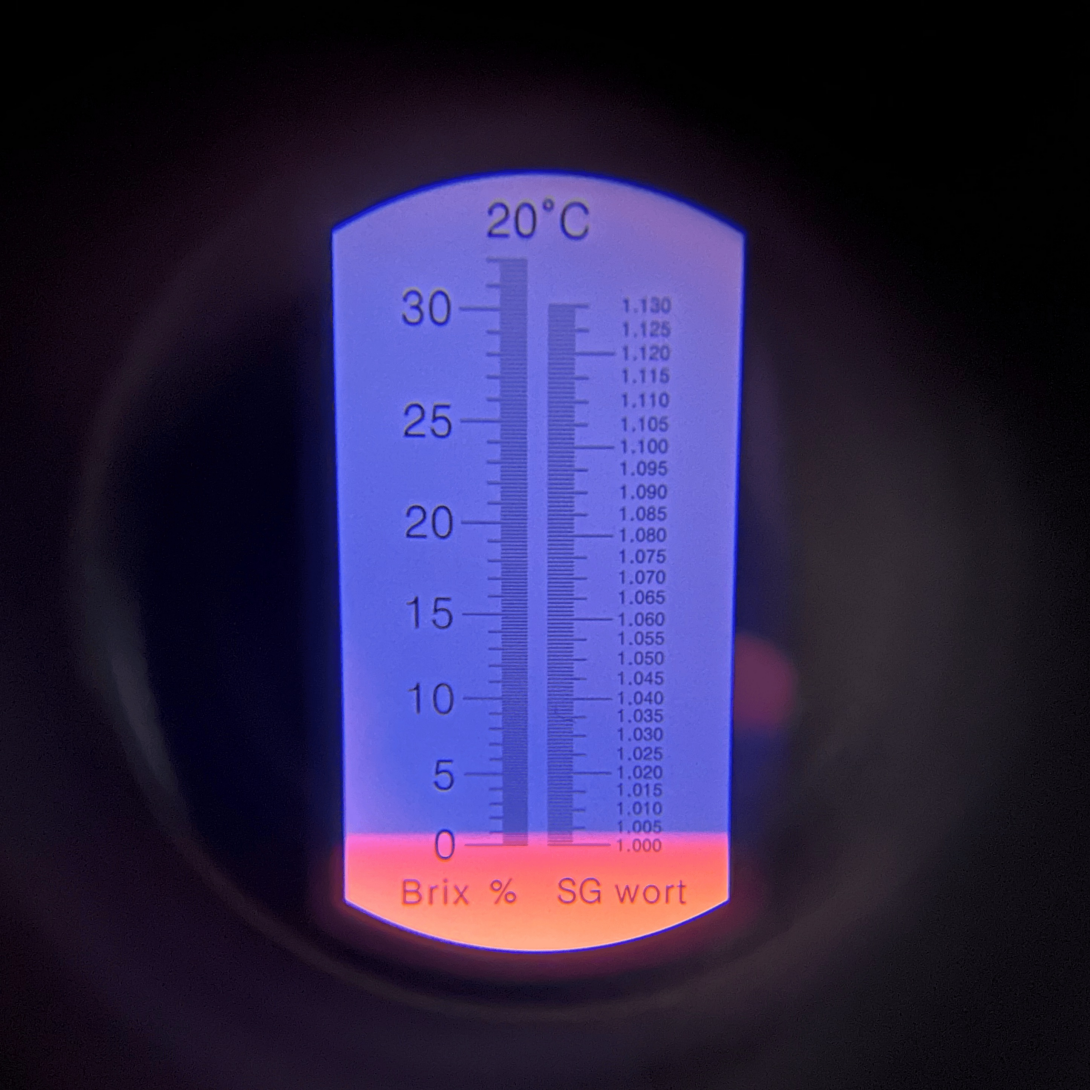
Refraction of solution minus cocoa powder showing specific gravity close to 1.005. Measured at 21 degrees Celsius. Image obtained with a specific gravity refractometer.

**Figure 9:**
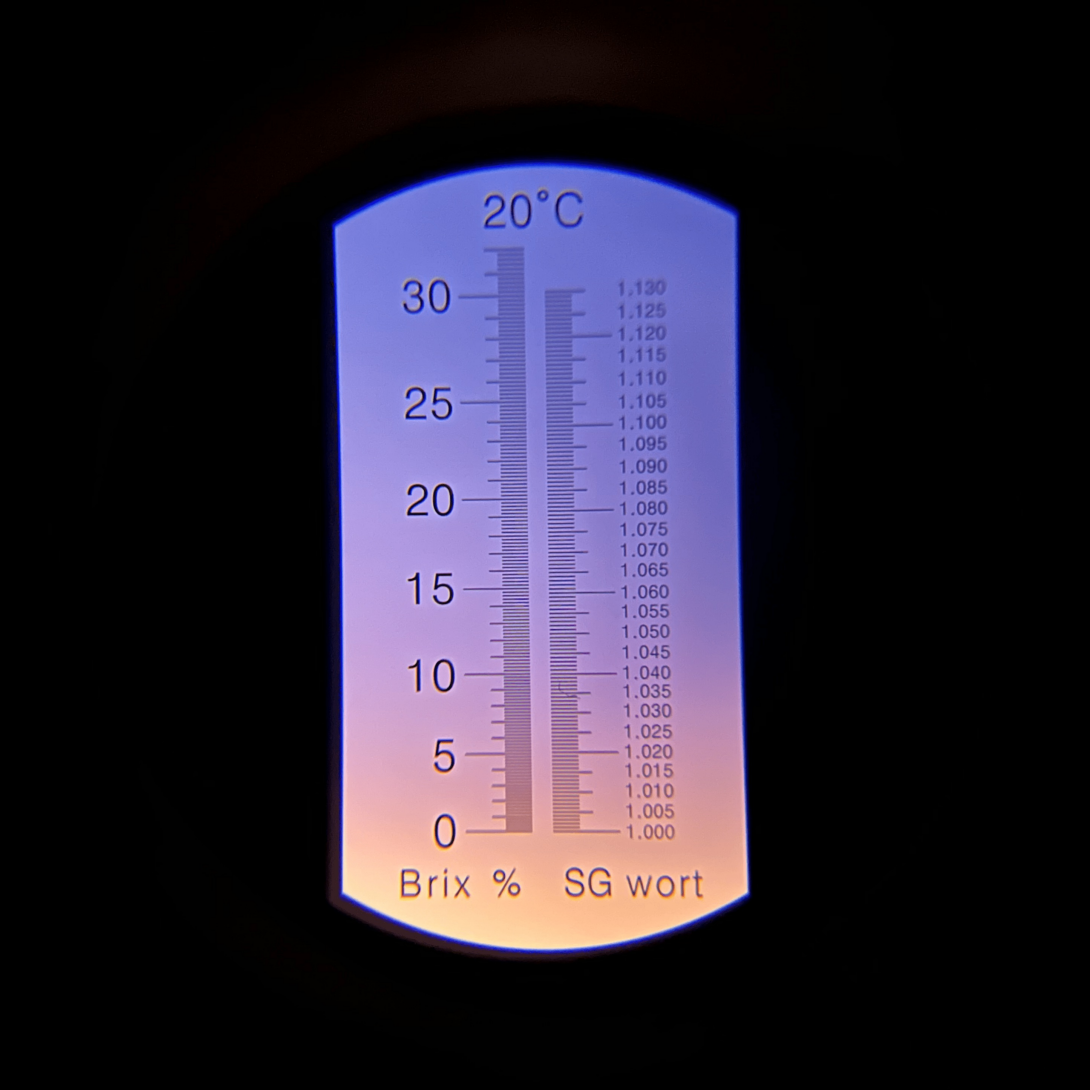
Refraction of final solution showing scattering of light. Measured at 21 degrees Celsius. Image obtained using a handheld refractometer. This image is included only as a visual demonstration of scatter. Because the cocoa suspension strongly scatters light, handheld refractometer readings were unreliable; density and specific gravity were not formally measured.

Each 1 L batch was prepared using room-temperature water, three single-serve packets of Crystal Light Sugar-Free Raspberry Ice Drink Mix, and 20 g of unsweetened cocoa powder. Water was placed into the mixing vessel first. Beverage powder was then added and blended for approximately 10-15 s. Cocoa powder was added gradually during continuous low-speed immersion blending to reduce clumping. Total blending time was approximately 30-60 s, or until no dry cocoa agglomerates were visible. The mixture was used immediately when possible. If held during a simulation session, the reservoir was covered and re-agitated or briefly re-blended before use and periodically during prolonged sessions because sedimentation was expected. Because the formulation contains organic material, it should be discarded after use; if temporary storage is necessary, it should be refrigerated in a closed container and re-homogenized before use. For this preparation, the immersion blender used was a Vitamix 5-Speed Immersion Blender (Olmsted Township, Ohio, USA) with a 625 W motor, operated on the fourth setting. Figures 6, 7 show single droplets of the solution, both with and without cocoa, to let the reader better visualize the gross visualization of the final mixture. Figures 8, 9 show refractometer-field images of the dye-only mixture and final cocoa-containing suspension. These images are included as qualitative demonstrations of altered light passage through the samples, not as validated density or optical performance measurements.

Preparation and handling considerations

Operationally, the most reliable preparation sequence is to first add water to a mixing vessel, then introduce the beverage-mix powder while agitating, followed by gradual addition of cocoa powder under continuous blending. This order reduces clumping and improves dispersion. Immersion blending for approximately 30 to 60 s typically produces a visually uniform suspension; additional blending can be used to increase uniformity but may entrain air bubbles, which can transiently alter appearance. If the simulant will be delivered via a pump circuit, the particulate load and viscosity should be verified against the pump’s specifications to reduce clogging risk, and inline filters should be avoided or sized appropriately.

For scenarios emphasizing optical obscuration rather than volumetric hemorrhage, the simulant can be introduced in smaller aliquots onto the oropharynx, esophagus, or endoscopic field to challenge lens clearing and suction technique. For scenarios requiring continuous flow (e.g., SALAD drills), periodic re-suspension in the reservoir is required to prevent progressive clearing as particulates settle. In manikin plumbing, cocoa particulates can accumulate at low-flow dead spaces; a post-session flush with warm water and detergent is advisable, followed by a clear-water rinse.

This formulation contains cocoa particulates and should not be introduced through the working channel of patient-care endoscopes or any device intended for later clinical use unless the institution’s endoscope-reprocessing team validates complete clearance for that specific device and workflow. High-level disinfection should not be assumed to remove retained organic particulate residue. For training use, the formulation is best suited to manikin plumbing, external lens fouling exercises, disposable/training scopes, or simulator channels that can be immediately flushed and visually inspected. If used in a training channel, we recommend immediate post-session flushing with warm water and detergent until the effluent is clear, followed by clear-water rinsing. Future work should include controlled channel-clearance testing using defined dwell time, flush volume, residue assessment, and reprocessing conditions.

Cost analysis

We performed a microcosting analysis from the perspective of a simulation program purchasing materials at retail list prices. Costs vary by retailer and contract pricing; we report list prices to support reproducibility and general estimates. Costs include only direct consumables (powders, premixed fluids) and exclude labor, shipping, storage, depreciation of pumps/manikins, and cleanup time. The proposed formulation’s unit costs were based on a representative 10-packet box price of $3.99 for the raspberry beverage mix and a representative 8 oz (227 g) container price of $4.92 for unsweetened cocoa powder [18,19]. Cocoa mass usage was estimated using a standard nutrition-label conversion of one tablespoon of cocoa powder, about 5 g (or 20 g per liter) [20]. Comparator product costs were obtained from manufacturer or distributor list prices for commonly used simulated blood premixes, powders, concentrates, and clotting blood simulants [12-17].

**Figure 10:**
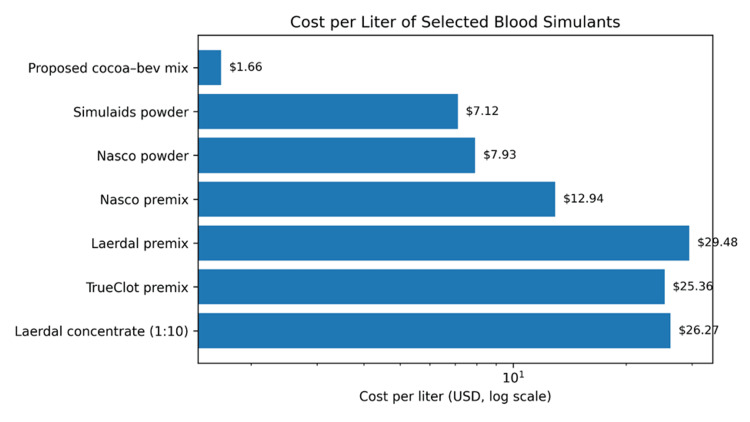
Demonstrates direct consumable cost per liter for the proposed formulation and selected commercially available simulated blood products. This is a cost comparison only; optical performance equivalence or superiority was not established in this preliminary report. Labels correspond to Table 2; log scaling is used to preserve readability across the $1-$30/L range. Credits: Kevin Malone. Created using Python (Python Software Foundation, Fredericksburg, VA, USA).

Using these inputs, the proposed cocoa-beverage mix costs at most approximately $1.66 per liter. In contrast, common commercial powders were approximately $7-$8 per liter, gallon-scale premixes approximately $13 per liter, and quart-scale premixes approximately $29 per liter. Concentrated blood products diluted to a light 1:10 ratio remained approximately $26 per liter, with substantially higher costs at darker dilutions [12-17]. At 100 L of annual consumption (a plausible order of magnitude for multi-cohort contaminated airway curricula), the proposed formulation would be approximately $166 in direct consumables, compared with approximately $700-$3,000 for typical commercial alternatives (exclusive of shipping). A microcosting comparison of the proposed formulation and selected commercial blood simulants is summarized in Table 2, and the order-of-magnitude spread in per-liter costs is illustrated in Figure 10.

**Table 2:** Microcosting comparison of selected blood simulants (retail list prices). Credits: Compiled by Kevin T. Malone. Data derived from manufacturer/distributor retail list prices and label-based ingredient conversion assumptions [12-20].

Formulation/product	Package/yield (USD)	Cost per liter (USD/L)	Cost for 100 L (USD)	Comments
Proposed cocoa-beverage mix	$3.99/10 packets + $4.92/227 g cocoa (retail)	Approximately 1.66	Approximately 166	Qualitatively opaque particulate suspension in bench photographs; not objectively benchmarked; may sediment and separate.
Simulaids simulated blood powder (225)	$26.95 per pack; makes 1 gallon (3.785 L)	7.12	711.94	General-purpose simulated blood; viscosity/opacity unspecified.
Nasco simulated blood powder (800-225)	$30.00 per pack; makes 1 gallon (3.785 L)	7.93	792.52	General-purpose simulated blood; manufacturer warns some staining.
Nasco simulation blood premix (LF00846)	$49.00 per gallon (3.785 L)	12.94	1,294.44	Ready-to-use; intended for skills trainers (IV/venipuncture).
Laerdal simulated blood premix (VT-491)	$27.90 per quart (0.946 L)	29.48	2,948.16	Marketed as stain-resistant and “same viscosity as real blood.”
TrueClot blood simulant premix	$95.99 per gallon (3.785 L)	25.36	2,535.79	Designed to clot with training gauze/solution; stable storage.
Laerdal concentrated blood (100019), 1:10 dilution	$28.90 per 100 mL; yield ~1.1 L at 1:10	26.27	2,627.27	Cost varies with dilution (manufacturer notes 1:2-1:10).

## Discussion

This preliminary report addresses a narrow but recurrent failure mode in simulation: inadequate optical obscuration. When the instructional objective is to stress-test visualization and suction technique, a simulant that remains translucent under LED illumination can unintentionally train learners to expect a view that is not available in real hematemesis. Based on formulation rationale and qualitative bench inspection, particulate-driven turbidity appears to be a plausible mechanism for increasing visual obscuration in these scenarios. However, this report did not quantify turbidity, optical density, spectral transmittance, camera occlusion, or learner-facing realism. The proposed cocoa-beverage mix leverages two complementary mechanisms - chromophore-based color and particle-based scattering - to approximate the perceptual appearance of hematemesis while remaining inexpensive, replicable, and made from materials commonly available outside specialized simulation supply chains.

Cost is not merely an administrative detail; it shapes what curricula are feasible. Large-volume “soiling” curricula are increasingly common in emergency medicine and critical care training, but commercial concentrates and premixes are priced in a way that discourages repetition. This constraint is amplified when programs attempt to scale training across interprofessional cohorts or across multiple clinical sites. Even non-blood contaminants used in airway simulation can be expensive at list price, which further increases the marginal cost of high-fidelity contaminated airway cases [21]. By reducing direct consumables to the $1-$2/L range, grocery-grade formulations can make high-repetition deliberate practice financially tractable, especially for resource-limited programs and for multi-center research protocols that require consistent materials.

The formulation’s principal limitation is physical stability. Sedimentation and phase separation are expected for a cocoa-based suspension because particle density exceeds that of water and the continuous phase viscosity is modest. Additionally, particulates are small, whereas in GI bleed, often particulates such as clots or vomitus are much larger. While re-blending/agitation solves the operational problem in most simulation settings, future optimization could explicitly target suspension stability (e.g., controlled particle size distribution and addition of low concentrations of food-grade thickeners to increase viscosity and introduce shear thinning). Such modifications should be evaluated against practical constraints, including pump compatibility, suction performance, residue in manikin plumbing, and cleanup burden. In an effort to keep the cost low and ease of access high, this recipe was made using two very common ingredients.

A second limitation is material compatibility. Staining risk depends on polymer composition, surface finish, porosity, and exposure time. The appropriate stance is empirical: each program should spot-test on the specific manikin and accessory materials in use and adopt a cleaning protocol that is effective without degrading the simulator. These considerations are not unique to this formulation; manufacturer instructions for commercial powders also warn that simulated blood can leave permanent stains on some surfaces [14]. In preliminary testing, the mixture appears to not stain silicone-based material; however, formal testing is needed.

A third limitation is the lack of formal objective data. Further formal testing of this solution in comparison to other readily purchasable simulants is needed. That stated, a majority of the solutions that exist are not created to obscure camera viewing, and therefore, we believe this formulation may be a possible improvement. Still, more formal testing is needed and is planned in the future.

This report is intentionally pragmatic and training-focused and should be interpreted as a preliminary formulation plus microcosting analysis rather than a validated blood-equivalence study. The next step is a standardized characterization workflow that quantifies optical performance under representative videolaryngoscope and endoscope illumination (e.g., spectral transmittance/reflectance or turbidity as a proxy for scattering), rheology across relevant shear rates and temperatures with attention to lens-film persistence, and density for pump calibration and flow reproducibility [8]. Because adoption in simulation centers is ultimately constrained by material compatibility, a formal staining and residue protocol should also be reported using controlled dwell and cleaning cycles across common manikin substrates (silicone, polyvinyl chloride (PVC), acrylonitrile butadiene styrene (ABS), polycarbonate, and acrylic) and optical coatings, consistent with existing manufacturer cautions that some simulated blood products can permanently stain selected materials [14]. Finally, to establish educational utility beyond face validity, randomized simulation studies should compare this hematemesis analog with representative commercial products using task-relevant endpoints such as time-to-clear-view, first-pass success, suction choreography quality, and cognitive load in contaminated airway and UGIB scenarios [1-6,11].

The principal limitation of this report is that it is a formulation proposal and microcosting analysis, not a validated blood-equivalence or superiority study. We did not measure nephelometric turbidity unit (NTU), spectral transmittance/absorbance, optical density, viscosity, shear-thinning behavior, density, standardized camera occlusion, clinician realism ratings, or direct performance against commercial simulants. Therefore, the data do not establish that this formulation is superior to commercial simulated blood products or equivalent to human blood or hematemesis. The contribution is a reproducible, low-cost, particulate recipe and rationale for future characterization in an area where published optical benchmarks for manikin-based blood simulants remain limited.

## Conclusions

For manikin-based training where realistic degradation of visualization is desired, this report proposes a low-cost cocoa-opacified beverage-powder hematemesis analog. The formulation is inexpensive and accessible and produces qualitative visual opacity in bench photographs, but optical or rheologic superiority over commercial products and equivalence to human blood were not established. Based on representative retail prices, the direct consumable cost was approximately $1.66/L, lower than the selected commercial simulated blood products reviewed here; this should be interpreted as a cost comparison only, not a performance comparison.

Programs adopting this formulation should anticipate sedimentation, re-homogenize before use, spot-test simulator materials, and avoid use in patient-care endoscope channels unless local reprocessing compatibility has been validated. Future studies should quantify turbidity, spectral transmittance/absorbance, viscosity, density, staining/residue, channel clearance, and standardized laryngoscope/endoscope performance compared with commercial simulants and, where feasible, blood.
